# Oral and intravenous pharmacokinetics of taurine in sprague‐dawley rats: the influence of dose and the possible involvement of the proton‐coupled amino acid transporter, PAT1, in oral taurine absorption

**DOI:** 10.14814/phy2.13467

**Published:** 2017-10-16

**Authors:** Carsten Uhd Nielsen, Maria Bjerg, Nithiya Ulaganathan, René Holm

**Affiliations:** ^1^ Department of Physics, Chemistry and Pharmacy University of Southern Denmark Odense M Denmark; ^2^ Pharmaceutical Science and CMC Biologics H. Lundbeck A/S Valby Denmark; ^3^ Present address: Drug Product Development Janssen R&D, Johnson & Johnson Beerse Belgium

**Keywords:** Intestinal absorption, PAT1, pharmacokinetics, Sprague‐Dawley rats, Taurine, TauT

## Abstract

Taurine is involved in various physiological processes, and one of the most abundant amino acids in human. The aim was to investigate the mechanism for intestinal absorption of taurine in vivo using also in vitro mechanistic studies. Taurine absorption was measured in male Sprague‐Dawley rats at 10–997 mg/kg and 1–30 mg/kg for oral and intravenous administration, respectively. Oral absorption was measured in the presence of substrates for the proton‐coupled amino acid transporter, PAT1, that is, 200 mg/kg proline (Pro) and sarcosine (Sar), and in the presence of 2‐Amino‐2‐norbornanecarboxylic acid (BCH) (200 mg/kg). BCH is not an inhibitor of PAT1 or the taurine transporter, TauT, hence it was included as a negative control. In vitro studies investigating the transport mechanism of taurine were conducted in human intestinal Caco‐2 cells. The pharmacokinetic investigations showed that intestinal taurine absorption was not saturable at the investigated doses, but that the time (*t*_max_) to reach the maximal plasma concentration (*C*_max_) increased with dose. Furthermore, Sar and Pro, but not BCH, decreased taurine *C*_max_. In vitro it was clearly shown that PAT1 mediated the cellular uptake of taurine and thereby facilitated the transepithelial taurine transport, which could be inhibited by Pro and Sar, but not BCH. In vivo and in vitro results suggest that taurine absorption from the intestine is caused by PAT1.

## Introduction

Taurine, 2‐aminoethane sulfonic acid, is involved in several biological processes; for example, serving as a precursor for the tauro‐conjugated bile salts, for example, taurocholate; for cellular volume regulation (for references see the excellent review by Huxtable (1992)) and; being important for retinal homeostasis through its function as an antioxidant and osmolyte (Huxtable 1992) (Ripps and Shen 2012). Taurine is available though both exogenous or endogenous sources. The exogenous source comes from oral ingestion of food, it can be found in large amounts in, for example, meat (Huxtable 1992). Taurine is also a component of infant formula and total parental nutrition for infants and adults. Moreover, taurine is currently a popular supplement in “high energy” drinks. These drinks contain an average of 3.18 g/L taurine (Caine and Geracioti 2016), with popular brands typically containing 4 g/L of taurine (Caine and Geracioti 2016), corresponding to an initial concentration of 32 mmol/L taurine which enters the gastrointestinal tract.

For many years, it has been known that the sodium‐chloride coupled taurine transporter, TauT, transports taurine across plasma membranes in a symport manner and the responsible transporter was cloned from various species in the early 1990s (Smith et al. 1992; Uchida et al. 1992; Jhiang et al. 1993). The taurine transporter is a high‐affinity transporter with *μ*M affinity for taurine and a low transport capacity (Liu et al. 1992; Smith et al. 1992). However, another intestinal amino acid transporter, the proton‐coupled amino acid transporter, PAT1 (SLC36A1), has also been suggested to facilitate taurine transport across intestinal cells (Thwaites et al. 1995; Anderson et al. 2004, 2009). PAT1 has low affinities for its substrates including taurine, that is, with mM affinities in contrast to the *μ*M range observed for TauT, and a high transport capacity (Larsen et al. 2008; Nohr et al. 2014b). In 2009, Anderson et al. (2009) demonstrated in vitro*,* in intestinal Caco‐2 cells, that both TauT and PAT1 contributed to cellular uptake of taurine, with PAT1 being the major transporter at higher concentrations.

Despite the scientific and nutritional interest in taurine and taurine transporting solute carriers, knowledge on the involvement of solute carriers (SLC) in oral absorption of taurine in vivo is limited and so is knowledge of oral and intravenous (IV) taurine pharmacokinetics. To the best of our knowledge, pharmacokinetic studies are limited to a few, none addressing the involvement of solute carriers; one study has investigated taurine absorption in healthy male human volunteers after administration of one dose of 4 g taurine in a capsule formulation given to the subjects (Ghandforoush‐Sattari et al. 2010). In male Sprague‐Dawley rats, taurine pharmacokinetics have been investigated after intravenous administration of 20 mg/kg taurine (Tang et al. 2014), and in male and female Sprague‐Dawley rats after oral administration of 30 or 300 mg/kg taurine (Sved et al. 2007). Taurine (6 mg/kg) has also been given intravenous to beagle dogs as a single administration together with 0.6 mg/kg edaravone (Yu et al. 2013). While these studies form an initial understanding of taurine pharmacokinetics, no systematic investigation of dose‐dependent intravenous or oral pharmacokinetics is available. Furthermore, the role of PAT1 in mediating intestinal taurine absorption in vivo has, to the best of our knowledge, not been investigated before.

The aim of this study was therefore to investigate the pharmacokinetics of taurine in vivo after oral and intravenous administration of different taurine doses, and furthermore to investigate if the proton‐coupled amino acid transporter PAT1 is involved in the oral absorption of taurine in vivo. Since PAT1 knock‐out animals are not available, we used an inhibitor‐based approach as previously used in the investigations of the role of PAT1 in intestinal gaboxadol and vigabatrin absorption (Larsen et al. 2009; Broberg et al. 2012; Nohr et al. 2014a, 2015). This was supplemented with mechanistic investigation of TauT and PAT1 mediated transport in vitro using intestinal Caco‐2 cells. Collectively, the work presented here shows that oral absorption of taurine was not saturated in the dose range of 10–997 mg/kg, yet the time (*t*_max_) to reach the maximal plasma concentration (*C*_max_) increased with dose. Furthermore, Sar and Pro, but not BCH, decreased taurine *C*_max_. BCH was chosen since it is not a substrate of TauT or PAT1 in vitro and does not affect PAT1 function in vivo in rats (Nohr et al. 2014c), but is an efficient inhibitor of LAT1 and LAT2 (Soares‐da‐Silva and Serrao 2004). In vitro it was clearly shown, that PAT1 mediated the cellular uptake of taurine and thereby facilitated the transepithelial taurine transport, which could be inhibited by Pro and Sar, but not BCH. Based on in vivo and in vitro mechanistic studies we can support a role of PAT1 in intestinal taurine absorption in vivo.

## Material and Methods

### Materials

Caco‐2 cells were obtained from DSMZ (Deutsche Sammlung von Mikroorganismen und Zellkulturen) (Braunschweig, Germany). Cell culture plastic ware was obtained from Corning Life Sciences (Tewksbury, MA, USA). Taurine (Tau), L‐proline (Pro), 2‐Amino‐2‐norbornane‐carboxylic acid (BCH), sarcosine (Sar), Triton X‐100, 4‐(2‐hydroxyethyl)‐piperazine‐1‐ethanesulfonic acid (HEPES), 4‐Morpholineethanesulfonic acid (MES), imidazole‐4‐acetic acid (IAA) and sodium bicarbonate were all obtained from Sigma‐Aldrich (St. Louis, MO, USA). Hank's Balanced Salt Solution (HBSS) with calcium and magnesium was obtained from Gibco by Life Technologies (Paisley, UK). D‐[1‐^14^C]‐Mannitol (57.2 mCi/mmol), [2,2‐^3^H(N)]‐Taurine (19.1 Ci/mmol) were purchased from Perkin Elmer (Waltham, MA, USA). Scintillation liquid was either OptiPhase Supermix cocktail (for in vivo studies) from Perkin Elmer (Waltham, MA, USA) or Ultima Gold (for in vitro studies) also from Perkin Elmer (Boston, MA, USA). Ethanol (96%) was from either Merck (Darmstadt, Germany) or VRW chemicals (Darmstadt, Germany). Purified water was used for the experiments, obtained from either a Milli‐Q and Elga purification system.

## Methods

### Cell culture

Polycarbonate membranes (1.12 cm^2^, 0.4 *μ*m pore size) of Transwell™ inserts or 24‐well cell culture plates (1.90 cm^2^) were used as supports for cell cultivation. The Caco‐2 cells were seeded at a density of 1.7 · 10^5^ cells cm^−2^. Experiments were performed 6 days after seeding the Caco‐2 cells cultured on 24 wells plates or performed 13–15 days postseeding cells on Transwell™ insert. The cells were maintained at 37°C in Dulbecco's modified Eagle medium (DMEM) supplied with 10% Fetal Bovine Serum (FBS), penicillin/streptomycin (10,000 U·mL^−1^/10 mg·mL^−1^), L‐glutamine (1%), and NonEssential Amino Acids (1%) in an atmosphere of 5% CO_2_ and a 90% relative humidity. The culture medium was changed every 2–3 days.

### Transport study buffer solutions

The donor solutions were prepared in Hanks Balanced Saline Solution (HBSS) buffered to either pH 7.4 or 6.0 using 10 mmol/L HEPES or MES, respectively. HBSS buffer consisted of in mM: CaCl_2_, 1.26; MgCl_2_, 0.49; MgSO_4_, 0.41; KCl, 5.33; KH_2_PO_4_, 0.44; NaCl, 138; Na_2_HPO_4_, 0.34; D‐glucose, 5.56; NaHCO_3_, 4.17 and contained 10 mmol/L MES at pH 6.0 (HBSS 6.0) or 10 mmol/L HEPES pH 7.4 (HBSS 7.4). The pH of the buffers was adjusted with NaOH/HCl using a pH‐meter (Mettler Toledo, FiveEasy™FE20, Schwerzenbach, Switzerland). Receiver solutions were HBSS pH 7.4 supplemented with various amount of NaCl to make the donor and receiver solution iso‐osmotic. Transport buffers with final concentration and osmolarity of BCH (128.9 mmol/L, 370 ± 30 mOsm), Pro (173.7 mmol/L, 430 ± 34 mOsm), Sar (224.5 mmol/L, 478 ± 40 mOsm) or a control were prepared with either HBSS pH 7.4 (280 ± 15 mOsm) or HBSS pH 6.0 (261 ± 28 mOsm). All donor solutions contained 1 *μ*Ci/mL [^3^H]‐taurine (19.1 Ci/mmol, 52 nmol/L) and the solutions prepared in HBSS pH 6.0 contained additionally 0.1 mmol/L nonradiolabeled taurine. Donor solutions also contained 1 *μ*Ci/mL [^14^C]‐mannitol (57.2 mCi/mmol, 17.5 *μ*mol/L) to measure the paracellular transport. The osmolality was measured with a Semi‐micro Osmometer K‐7400 (Knauer, Berlin, Germany), and the receiver solutions were adjusted with NaCl to make them iso‐osmotic with the corresponding donor solutions.

### Taurine uptake in Caco‐2 cells

The uptake of taurine was investigated at either pH 6.0 or pH 7.4 for 5 min to assess the contribution of TauT and PAT1 to the total taurine uptake. Previous studies have shown that uptake of PAT1 substrates in Caco‐2 cells is linear for 5 min (Larsen et al. 2008; Nohr et al. 2014b). The concentration‐dependent taurine (1 *μ*Ci/mL [^3^H]‐taurine, 52 nmol/L) uptake at pH 7.4 was measured at total taurine concentrations of 0.052, 5, 10, 25, 50, and 100 *μ*mol/L in HEPES buffer, pH 7.4. Taurine concentrations of 0.052, 0.1, 2, 5, 10, 25, and 50 mmol/L in MES buffer were investigated at pH 6.0. The uptake of 52 nmol/L taurine (1 *μ*Ci/mL [^3^H]‐taurine) at pH 7.4 was also measured in the presence of 20 mg/mL BCH, 20 mg/mL Sar, 10 mmol/L imidazole‐4‐acetic acid (IAA), which was recently identified as a ligand of TauT (Valembois et al. 2017), and 20 mg/mL Pro. The taurine (0.1 mmol/L, 1 *μ*Ci/mL [^3^H]‐taurine) uptake at pH 6.0 was likewise measured in the presence of 20 mg/mL BCH, 20 mg/mL Sar or either 10 mmol/L of Sar, Pro, BCH, and IAA.

After the 5 min incubation, the isotope‐containing buffer solution was removed and the wells were washed three times with 500 *μ*L ice‐cold HEPES‐buffer, pH 7.4. The cells were detached using 200 *μ*L of 0.1% Triton X‐100, and transferred to scintillation plastic vials and 2 mL of scintillation cocktail was added. The samples were vortexed 10 s and DPM was measured for 10 min using a liquid scintillation counter (LSC) (Perkin Elmer, Tri‐Carb 2900TR).

### Transport of taurine across Caco‐2 cell monolayers

Caco‐2 cells grown on Transwell^®^ filters (1.12 cm^2^, 0.4 *μ*m) were used for the transport study. The initial transepithelial electrical resistance (TEER) was measured to assure that the barrier properties of the monolayer were intact. Transport was measured from the apical (A) to the basolateral (B) side. Before starting the experiment, all solutions were preheated to a temperature of 37°C. The cells were incubated with prewarmed HBSS 7.4 for at least 10 min on a mini shaker (220 rpm) from Troemner (NJ, US) at 37°C to equilibrate the cells prior to the experiment. Buffer was removed and the appropriate solutions were applied on the apical and basolateral side in a volume of 500 *μ*L and 1000 *μ*L, respectively. Samples, in a volume of 100 *μ*L, were taken from the receiver solution at 15, 30, 60, 90 and 120 min after starting the experiment and replaced with 100 *μ*L fresh HBSS 7.4 on the basolateral side. Donor samples were taken from each solution containing radiolabeled compound in a volume of 20 *μ*L at 0 and 120 min. All samples were transferred directly into scintillation vials. After the experiment, the TEER value of each monolayer was measured again. To all samples, 2 mL scintillation liquid was added and vortexed for 10 sec. The amount of radiolabeled compound (as disintegration per minute, DPM) was analyzed for 10 min using liquid scintillation counter (LSC) (Perkin Elmer, Tri‐Carb 2900TR).

### Formulations for the pharmacokinetic study

Taurine, BCH, proline, and sarcosine were dissolved in Elga water. Tonicity of the control solution was adjusted with sodium chloride to approximately 280 mOsmol/L. The osmolality of the formulation containing BCH was 326 mOsmol/L and the solution containing Pro and Sar had an osmolality of 394 mOsmol/L. Osmolalities were measured using a vapor pressure osmometer by Wescor Vapro model 5520 (Wescor Inc, Logan, Utah, USA). pH values were measured using PHM220 LAB pH meter by Radiometer analytical SAS (Lyon, France). The pH was adjusted with NaOH or HCl to 7.40 ± 0.05. Immediately before dosing, [^3^H]‐taurine was added to each formulation containing taurine in an activity of 40 *μ*Ci/mL.

### Taurine pharmacokinetics in Sprague‐Dawley rats

Male Sprague‐Dawley rats, weighing approximately 350 g (287–398 g) on the day of the experiments, were purchased from Charles River Deutschland (Sulzfeld, Germany). The animals were acclimatized for a minimum of 5 days in groups of two and maintained on standard feed with free access to water. Prior to dosing, the animals were fasted for 16–20 h with free access to water. The protocol used for the animal studies was approved by the institutional animal ethics committee in accordance with Danish law regulating animal experiments and in compliance with EC Directive 2010/63/EU and the NIH guidelines on animal welfare.

Five groups of four male Sprague‐Dawley rats were dosed with taurine solutions (10, 30, 100, 299, 997 mg/kg, with 40 *μ*Ci/kg [^3^H]‐taurine) orally by gavage (10 mL/kg). Additionally, three groups of four male Sprague‐Dawley rats were dosed with taurine solutions (1, 10, 30 mg/kg) intravenously by injection (5 mL/kg) into the tail vein. Another group of rats was dosed with a formulation containing 30 mg/kg taurine (with 40 *μ*Ci/kg [^3^H]‐taurine) and 200 mg/kg BCH orally by gavage (10 mL/kg). The last group was dosed with a formulation containing 30 mg/kg taurine, 200 mg/kg Pro and 200 mg/kg Sar orally by gavage (10 mL/kg).

### Plasma sampling and sample analysis

The blood samples (100–200 *μ*L), collected after administration, were drawn from the tail vein and collected into EDTA coated tubes (Microwette 500 K3E, Sarstedt, Nümbrecht, Germany) at 5–360 min after administration (5, 15, 30, 45, 60, 120, 180, 240, 360 min). Plasma was harvested immediately by 10 min of centrifugation at 4°C, 3600*g* (Multifuge 1 S‐R, Heraeus, Hanau, Germany) and stored in polypropylene tubes (Pony Vial, PerkinElmer, Waltham, MA, USA) at −20°C until analysed. At the end of the experiment, animals were sacrificed by spinal dislocation using a guillotine. Plasma samples were left to defrost and mixed with 4 mL of liquid scintillation cocktail. The samples were counted for 3 min on a scintillation counter from Tri‐Carb 2900TR.

## Data analysis

Transport data was described by Fick′s first law;

$$J=\frac{m}{A\cdot t}=P_{\mathrm{app}}\cdot C_{0}$$

where m is the steady‐state amount of transported compound over a given absorptive area (A), J is the flux, *t* is time and C_0_ is the starting concentration in the donor chamber. From the steady‐state flux the apparent permeability coefficient (*P*_app_) was calculated.

The total uptake of taurine at pH 6.0 and 7.4 in Caco‐2 cells was described by Michaelis‐Menten like kinetics with a nonsaturable component:

$$V_{o}=\frac{V_{\max}\cdot \left[\mathrm{Tau}\right]}{K_{m}+\left[\mathrm{Tau}\right]}+B\cdot \left[\mathrm{Tau}\right]$$

where *V*_o_ is the initial uptake rate of taurine at a given [Tau], which is the concentration of taurine in mmol/L or *μ*mol/L in the donor solution, *V*_max_ is the maximal uptake rate, *K*_m_ is the Michaelis constant, and B is a factor correcting for nonspecific binding of the isotope and for isotope dilution.

The total carrier‐mediated taurine uptake rate at pH 6.0 was described by:

$$V_{o}=\frac{V_{\max,\mathrm{PAT}1}\cdot \left[\mathrm{Tau}\right]}{K_{m,\mathrm{PAT}1}+\left[\mathrm{Tau}\right]}+\frac{V_{\max,\mathrm{TauT}}\cdot \left[\mathrm{Tau}\right]}{K_{m,\mathrm{TauT}}+\left[\mathrm{Tau}\right]}$$

The pharmacokinetic parameters of intravenously bolus administered taurine in male Sprague‐Dawley rats were estimated using a two‐compartmental model with elimination from the central compartment:

$$C\left(t\right)=A\cdot e^{-\alpha t}+B\cdot e^{-\beta t}$$

where C(t) is the taurine plasma concentration at a given time point, and A and *α* and B and *β* are hybrid constants related to the concentration in the central and peripheral compartments, respectively, and the rate of transfer between these. Based on this *V*_d1_, volume of distribution in the central compartment 1; *V*_d2_, volume of distribution in the peripheral compartment 2; AUC, area under the curve; and K10, the elimination rate from the central department, were calculated using Phoenix^®^ 6.3 version (Pharsight Corporation, A Certara Company, USA). The pharmacokinetic parameters of orally administered taurine in male Sprague‐Dawley rats were estimated noncompartmentally using Phoenix^®^ 6.3 version (Pharsight Corporation, A Certara Company, USA). *C*_max_ and *t*_max_ were found as mean values of the plasma concentration profiles within each group. Area under the plasma concentration versus time profiles (AUC), an expression of total exposure, were determined with noncompartmental analysis using the linear trapezoidal rule from time zero to *C*_max_ and by log linear methods from *C*_max_ to the last measured plasma concentration and denoted AUC_0‐tlast_, *k*_e_ is the first order elimination rate constant calculated as the slope from the terminal log plasma concentration time curve of individual animals. The total clearance was calculated as dose divided by AUC.

## Statistical analysis

Statistical analysis was performed in GraphPad Prism version 7.02. The obtained data was analysed for statistical differences using Nonparametric Kruskal–Wallis followed by Dunn's multiple comparison test. The following levels of significance were assigned: nonsignificant (NS) and *P* < 0.05 (*). However, for convenience values were expressed as mean ± SEM.

## Results

### Concentration‐dependent taurine uptake in Caco‐2 cells under neutral and slightly acidic conditions

**Figure 1:**
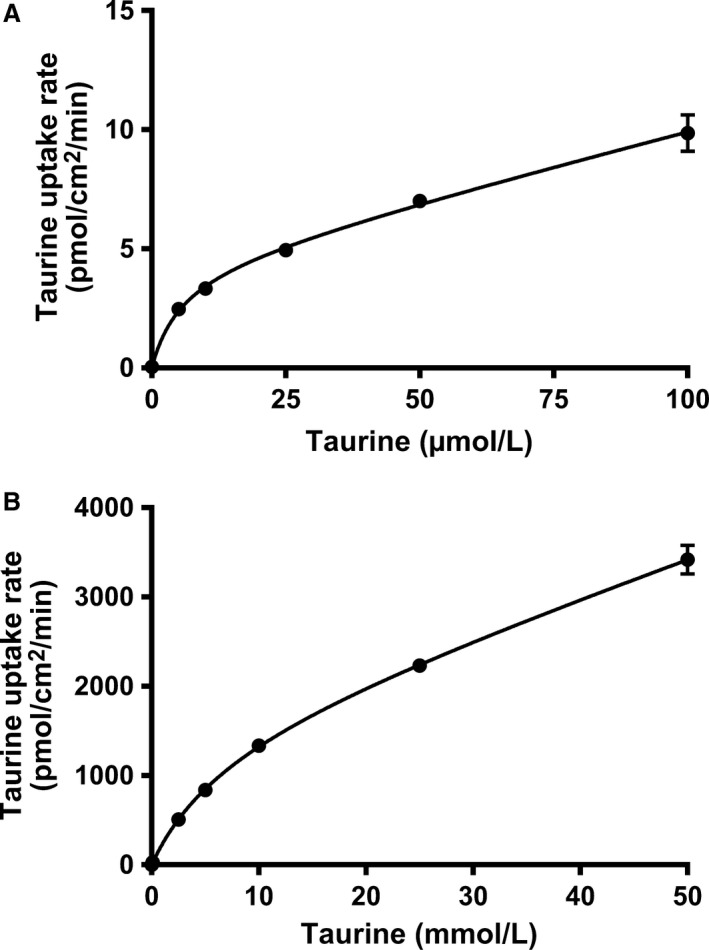
Concentration‐dependent cellular uptake of [^3^H]‐taurine in Caco‐2 cells. (A) The uptake rate of taurine in Caco‐2 cells at 37°C in the presence of increasing concentrations of unlabelled taurine. The buffer used was HBSS containing 10 mmol/L HEPES adjusted to pH 7.4. (B) The buffer used was HBSS containing 10 mmol/L MES adjusted to pH 7.4. The uptake was measured for 5 min. The concentration of [^3^H]‐taurine was 1 *μ*Ci·mL ^−1^ (52 nmol/L). Each value represents the mean ± SEM of four independent cell passages (*n* = 4). The solid lines show the fit of the resulting data to Michaelis‐Menten like kinetics.

To investigate the underlying cellular mechanism for taurine absorption a series of in vitro experiments were conducted in the human intestinal Caco‐2 cell model. At first the uptake was measured with neutral (pH 7.4) and slightly acidic (pH 6.0) buffer solutions. When the extracellular pH is 7.4 only TauT is mediating taurine uptake, while when pH is 6.0 both PAT1 and TauT mediate cellular taurine uptake. The uptake of taurine was measured at neutral pH conditions at the concentration range of 0.052–100 *μ*mol/L (see Fig. 1A). The uptake was described by a saturable and nonsaturable process. The saturable process was described by a *K*_m_‐value of 5.6 ± 2.6 *μ*mol/L and a *V*_max_ of 4.6 ± 0.8 pmol/(cm^2^·min), while the nonsaturable component, B, was 0.057·10^−6^ ± 0.008·10^−6^ cm/min. In the presence of a slight pH gradient (pH 6.0) (Fig. 1B), the saturable taurine uptake process was described by a *K*_m_‐value of 7.1 ± 2.3 mmol/L and a *V*_max_ of 1559 ± 309 pmol/(cm^2^·min), while the nonsaturable component, B, was 41·10^−6^ ± 5·10^−6^ cm/min.

### Taurine uptake in Caco‐2 cells in the presence of BCH, IAA, Pro, and Sar

**Figure 2:**
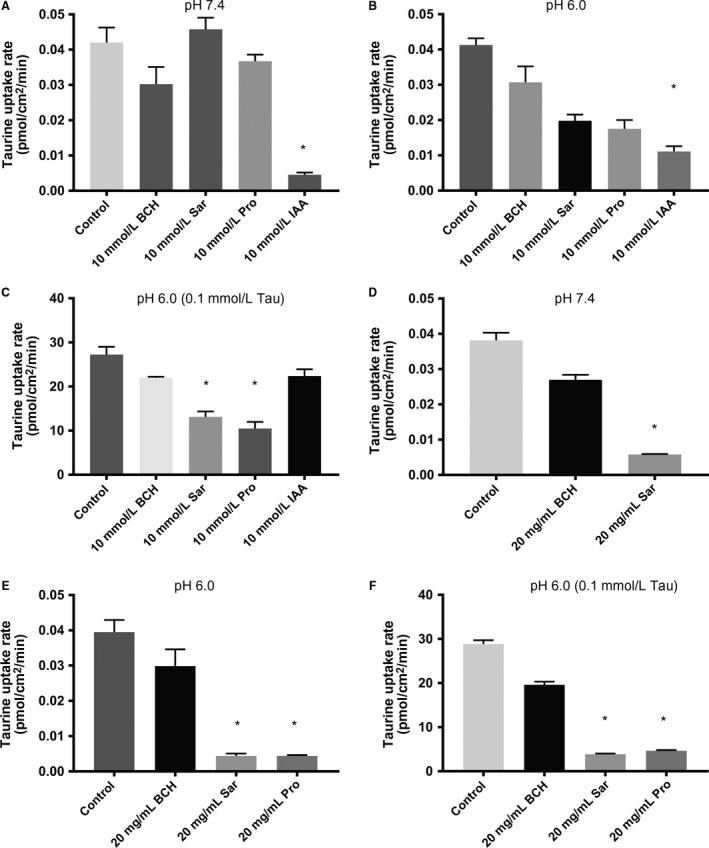
Cellular uptake of [^3^H]‐taurine in Caco‐2 cells in the presence of amino acids. (A). The uptake rate of taurine in Caco‐2 cells at 37°C in the absence or presence of 10 mmol/L amino acid. The buffer used was HBSS containing 10 mmol/L HEPES adjusted to pH 7.4. The concentration of [^3^H]‐taurine was 1 *μ*Ci·mL ^−1^ (52 nmol/L). (B). The uptake rate of taurine in Caco‐2 cells at 37°C in the absence or presence of 10 mmol/L amino acid. The buffer used was HBSS containing 10 mmol/L MES adjusted to pH 6.0. The concentration of [^3^H]‐taurine was 1 *μ*Ci·mL ^−1^ (52 nmol/L). (C) The uptake rate of taurine in Caco‐2 cells at 37°C in the absence or presence of 10 mmol/L amino acid. The buffer used was HBSS containing 10 mmol/L MES adjusted to pH 6.0. The concentration of [^3^H]‐taurine was 1 *μ*Ci·mL ^−1^ (52 nmol/L) supplemented with unlabeled taurine to a total concentration of 100 *μ*mol/L. (D) The uptake rate of taurine in Caco‐2 cells at 37°C in the absence or presence of 20 mg/mL amino acid. The buffer used was HBSS containing 10 mmol/L HEPES adjusted to pH 7.4. The concentration of [^3^H]‐taurine was 1 *μ*Ci·mL ^−1^ (52 nmol/L). (E) The uptake rate of taurine in Caco‐2 cells at 37°C in the absence or presence of 20 mg/mL amino acid. The buffer used was HBSS containing 10 mmol/L MES adjusted to pH 6.0. The concentration of [^3^H]‐taurine was 1 *μ*Ci·mL ^−1^ (52 nmol/L). (F) The uptake rate of taurine in Caco‐2 cells at 37°C in the absence or presence of 20 mg/mL amino acid. The buffer used was HBSS containing 10 mmol/L MES adjusted to pH 6.0. The concentration of [^3^H]‐taurine was 1 *μ*Ci·mL ^−1^ (52 nmol/L) supplemented with unlabelled taurine to a total concentration of 100 *μ*mol/L. For all conditions, the uptake was measured for 5 min. Each value represents the mean ± SEM of four to seven independent cell passages (*n* = 4–7). *:*P* < 0.05

The uptake of taurine was investigated in Caco‐2 cells at pH 7.4 and pH 6.0 at two different concentration, that is, 0.052 *μ*mol/L and 100 *μ*mol/L, used as conditions were TauT and PAT1, respectively, dominate cellular uptake of taurine. With a taurine concentration of 0.052 *μ*mol/L at pH 7.4, the taurine uptake was not affected by 10 mmol/L of the PAT1 substrates proline or sarcosine or the non‐PAT1 and non‐TauT substrate BCH, whereas the TauT inhibitor IAA significantly inhibited the uptake of a taurine to approximately 10% of the control uptake (Fig. 2A). At pH 6.0 with a donor concentration of taurine of 0.052 *μ*mol/L, the uptake of taurine was not affected by 10 mmol/L of BCH, and was significantly inhibited by 10 mmol/L IAA (Fig. 2B). Even though the uptake has approximately 50% of the control uptake in the presence of 10 mmol/L Sar and Pro this was not a significant reduction with the employed statistical analysis. When the uptake of taurine was measured at pH 6.0 at a higher taurine concentration of 100 *μ*mol/L, the uptake was not affected significantly by 10 mmol/L BCH or 10 mmol/L IAA (Fig. 2C). The uptake was, however, significantly decreased in the presence of 10 mmol/L Sar or 10 mmol/L Pro (Fig. 2C). The uptake of taurine was also measured in the presence of inhibitor amounts similar to the ones dosed in the animal studies, that is, 20 mg/mL under neutral condition (pH 7.4) Sar, but not 20 mg/mL BCH, decreased the uptake of 0.052 *μ*mol/L taurine (Fig. 5D). Under slightly acidic conditions, pH 6.0, 20 mg/mL Sar and Pro significantly inhibited the taurine uptake at both taurine concentrations (Fig. 2E and F), whereas 20 mg/mL BCH did not affect taurine uptake at neither 0.052 *μ*mol/L nor 100 *μ*mol/L (Fig. 2E and F).

### The contribution of PAT1 and TauT to the carrier‐mediated taurine uptake

Based on the data presented in the present work, PAT1 did not contribute to taurine uptake at pH 7.4 and the carrier‐mediated uptake at pH 7.4 was attributed to TauT. At pH 6.0 the uptake of taurine was attributed to PAT1, due to the concentration range investigated. The two Michaelis‐Menten like expressions were then combined to estimate the total carrier‐mediated taurine uptake at pH 6.0:

$$V_{\mathrm{tau}}=\frac{1559\frac{\mathrm{pmol}}{cm^{2}\cdot \min}\cdot \left[\mathrm{Tau}\right]}{7123\mu \mathrm{mol}/L+\left[\mathrm{Tau}\right]}+\frac{4.6\frac{\mathrm{pmol}}{cm^{2}\cdot \min}\cdot \left[\mathrm{Tau}\right]}{5.6\mu \mathrm{mol}/L+\left[\mathrm{Tau}\right]}$$

**Figure 3:**
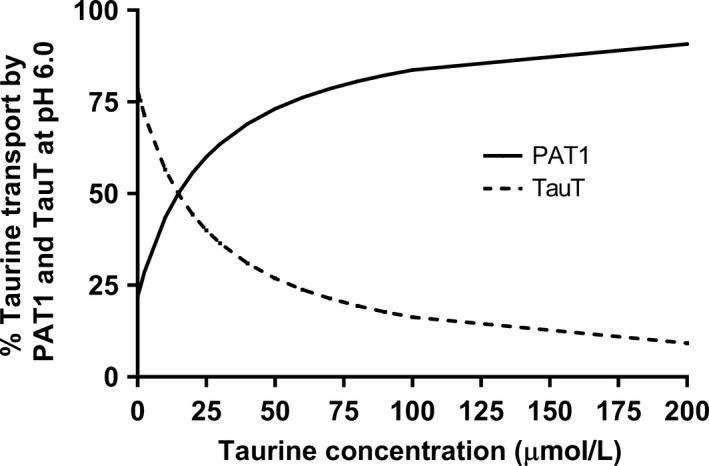
The estimated contribution of the taurine transporter (TauT) and the proton‐coupled amino acid transporter (PAT1) to carrier‐mediated cellular uptake of taurine at pH 6.0 in Caco‐2 cell monolayers.

In Figure 3 the estimated contribution of PAT1 and TauT to the carrier‐mediated uptake of taurine in Caco‐2 cells at pH 6.0 is shown in percentages. Based upon this, it could be estimated that at 52 nmol/L, TauT was responsible for 78% of the carrier mediated uptake, at 100 *μ*mol/L PAT1 was responsible for 84% of the carrier‐mediated taurine uptake, at 1 mmol/L 98% and, at 10 mmol/L 99.5%.

### Transepithelial transport of taurine across Caco‐2 cell monolayers

**Figure 4:**
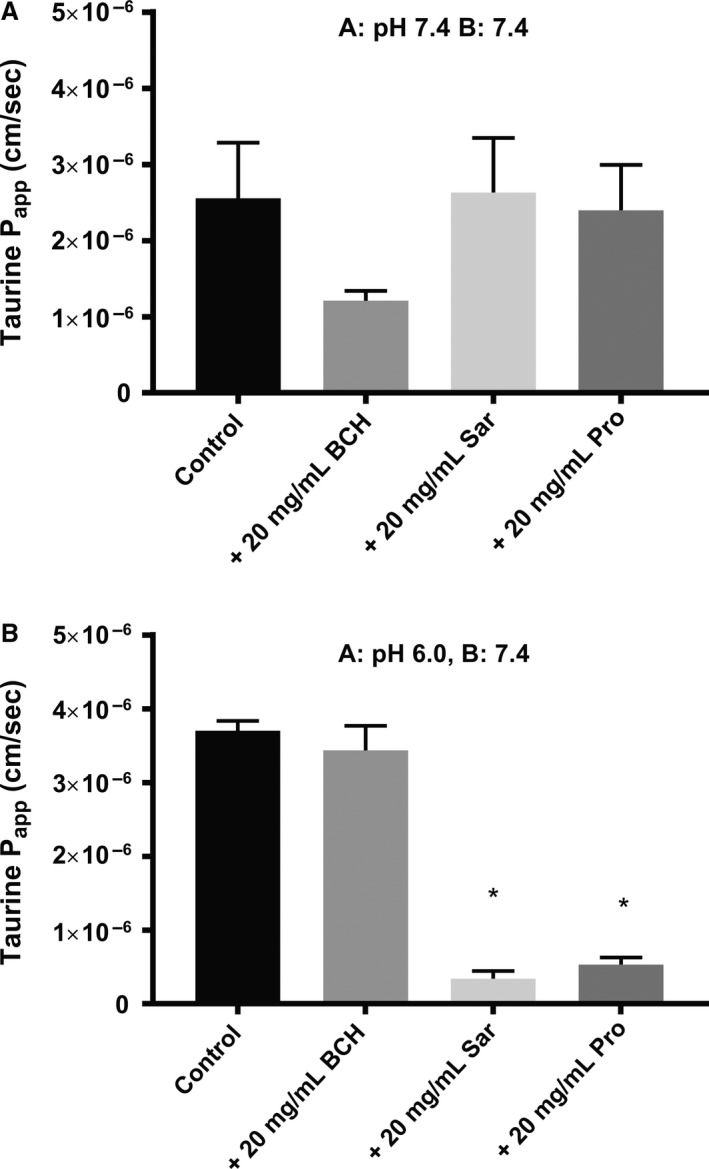
Transepithelial taurine across Caco‐2 cell monolayers in the presence of amino acids. The apparent transepithelial permeability, *P*_app_, of taurine across Caco‐2 cell monolayers from apical to basolateral side (A–B). The transepithelial transport was measured for 120 min and the permeability was calculated under steady‐state conditions from Ficks first law and expressed as mean ± SEM of measurements performed in three to four independent cell passages (*n* = 3–4). (A) Apical and basolateral pH was 7.4 for [^3^H]‐taurine (1 *μ*Ci/mL [^3^H]‐taurine, 52 nmol/L) permeability across Caco‐2 cell monolayers in the absence or presence of 20 mg/kg BCH (128.9 mmol/L), proline (173.7 mmol/L) or sarcosine (224.5 mmol/L). (B) Apical pH was 6.0 and basolateral pH was 7.4 for 100 *μ*mol/L taurine (1 *μ*Ci/mL [^3^H]‐taurine, 52 nmol/L) permeability across Caco‐2 cell monolayers in the absence or presence of 20 mg/kg BCH (128.9 mmol/L), proline (173.7 mmol/L) or sarcosine (224.5 mmol/L). *: *P* < 0.05

The transepithelial transport of taurine across Caco‐2 cell monolayers in the absorptive (A‐B) direction was investigated under conditions facilitating either TauT or PAT1 mediated transport. The transport of taurine at 0.052 *μ*mol/L in the presence of 20 mg/mL BCH, Pro and Sar is shown in Figure 4A. The presence of BCH, Sar or Pro did not significantly decrease the absorptive permeability of taurine. Under conditions facilitating PAT1 mediated transport, that is, at a donor taurine concentration of 100 *μ*mol/L and with a donor pH of 6.0, BCH did not alter the taurine permeability, while both Sar and Pro significantly decreased the absorptive taurine permeability, see Figure 4B. To investigate the barrier properties of Caco‐2 cell in the presence of high amino acid concentrations TEER values were measured at room temperature before and after the experiment and mannitol transport was evaluated during the transport experiment (Table 1). TEER values decreased during the transport experiment, most notable at neutral pH in the presence of 20 mg/mL Sar and Pro (Table 1). This decrease in TEER was also followed by an increase in mannitol permeability. However, at pH 6.0 in the apical chamber the different treatment did not alter mannitol transport.

**Table 1:** Apparent transepithelial permeability of [^14^C]‐mannitol and transepithelial electrical resistance (TEER) values of Caco‐2 cell monolayers

	A–B transport pH 7.4/7.4			A–B transport pH 6.0/7.4		
	Mannitol *P*_app_ (cm/s)	TEER (Ω·cm^2^)		Mannitol *P*_app_ (cm/s)	TEER (Ω·cm^2^)	
		Before	After		Before	After
Control	0.9 x 10^−7^ ± 9.3 x 10^−9^	541 ± 40	421 ± 31	1.1 x 10^−7^ ± 3.4 x 10^−8^	489 ± 49	472 ± 9
+ BCH (20 mg/mL)	1.6 x 10^−7^ ± 7.6 x 10^−9^	512 ± 40	299 ± 28	1.4 x 10^−7^ ± 1.3 x 10^−8^	491 ± 54	349 ± 57
+ Pro (20 mg/mL)	3.4 x 10^−7^ ± 7.5 × 10^−8^ (*)	486 ± 62	135 ± 23	1.7 x 10^−7^ ± 1.3 x 10^−8^	509 ± 64	354 ± 56
+ Sar (20 mg/mL)	3.2 x 10^−7^ ± 7.9 × 10^−8^ (*)	467 ± 48	171 ± 53	1.8 x 10^−7^ ± 1.6 x 10^−8^	514 ± 54	401 ± 69

The apparent transepithelial permeability, *P*_app_, of 1 *μ*Ci/mL [^14^C]‐mannitol (57.2 mCi/mmol, 17.5 *μ*mol/L) mannitol across Caco‐2 cell monolayers from apical to basolateral side (A–B) obtained under the conditions shown in Figure 7. The transepithelial transport was measured for 120 min and the permeability was calculated under steady‐state conditions from Fick′s first law and expressed as mean ± SEM of measurements performed in three to four independent cell passages (*n* = 3–4). A–B transport pH 7.4/7.4: Apical and basolateral pH was 7.4 for control ([^3^H]‐taurine (1 *μ*Ci/mL [^3^H]‐taurine, 52 nmol/L)) and the presence of 20 mg/kg BCH (128.9 mmol/L), proline (173.7 mmol/L) or sarcosine (224.5 mmol/L). A–B transport pH 6.0/7.4: Apical pH was 6.0 and basolateral pH was 7.4 for control (100 *μ*mol/L taurine, 1 *μ*Ci/mL [^3^H]‐taurine) and presence of 20 mg/kg BCH (128.9 mmol/L), proline (173.7 mmol/L) or sarcosine (224.5 mmol/L). *:*P* < 0.05.

### Intravenous administration of taurine to male Sprague‐Dawley rats

**Figure 5:**
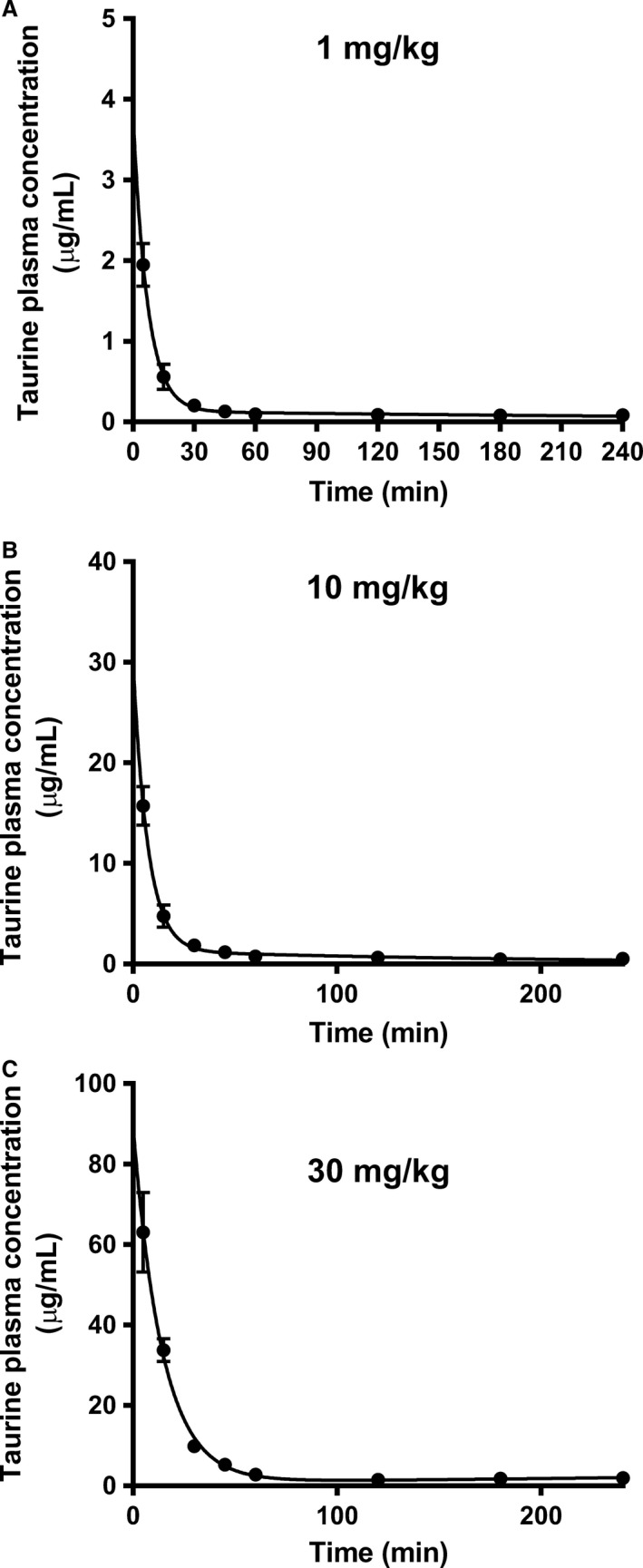
Taurine PK curves after IV administration to male Sprague‐Dawley rats. Taurine plasma concentration versus time profiles following intravenous administration of [^3^H]‐taurine (40 *μ*Ci/kg) to a total dose of 1.0 mg/kg (A), 10 mg/kg (B) or 30 mg/kg (C) taurine. The data was fitted to a two‐compartmental model $C\left(t\right)=A\cdot e^{-\alpha t}+B\cdot e^{-\beta t}$. Data is presented as mean ± SEM of measurements from 4 to 8 rats per dosing group.

Male Sprague‐Dawley rats were dosed by intravenous administration of taurine at three doses of 1, 10, and 30 mg/kg, and the taurine plasma concentration was monitored over 4 h (see Fig. 5). This gave measured plasma concentrations in the range of 0.1–63 *μ*gram/mL, equivalent to 8–504 *μ*mol/L (Fig. 5). The pharmacokinetic (PK) profiles were fitted to a two‐compartment model, and the obtained values for A, B, *α,* and *β* are given in Table 2 and the estimated pharmacokinetic parameters in Table 3. No differences were found between groups for *V*_d1_, *V*_d2_, K10 or AUC_0‐∞_ normalized for the dose of taurine. The relative exposure of taurine was thus similar in the measured plasma concentration interval and independent of the administered dose over the dose range investigated.

**Table 2:** Pharmacokinetic parameters for two‐compartment pharmacokinetics after intravenous administration of taurine at three doses of 1, 10, and 30 mg/kg to male Sprague‐Dawley rats

Taurine dose (mg/kg)	A (*μ*g/mL)	B (*μ*g/mL)	*α* (1/min)	*β* (1/min) *10^−3^
1	3.5 ± 0.5	0.12 ± 0.03	0.14 ± 0.02	1.9 ± 0.4
10	31.0 ± 4.2	0.89 ± 0.14	0.144 ± 0.03	3.0 ± 0.9
30	99.9 ± 15.5	1.87 ± 0.45	0.07 ± 0.01	1.3 ± 0.6

Data is presented as mean ± SEM of measurements from 4 to 6 rats per dosing group

**Table 3:** Pharmacokinetic parameters of taurine after intravenous administration to male Sprague‐Dawley rats

Taurine dose (mg/kg)	CL (mL/min/kg)	*V*_d1_ (mL/kg)	*V*_d2_ (mL/kg)	AUC_0‐∞_ (*μ*g·min/mL)	AUC_0‐∞_/dose (*μ*g·min/mL pr. mg/kg)	K10 (1/min)
1	11.7 ± 1.30	299 ± 48	4608 ± 974	89 ± 9	89 ± 18.9	0.04 ± 0.01
10	18.7 ± 2.54	353 ± 62	4662 ± 1153	587 ± 77	59 ± 19.0	0.06 ± 0.01
30	9.4 ± 1.42	323 ± 63	8374 ± 4466	3452 ± 588	115 ± 39.2	0.03 ± 0.01

Rats were intravenously administered 40 *μ*Ci/kg [^3^H]‐taurine with the total doses indicated. Data are expressed as mean ± SEM of measurements from 4 to 8 rats per dosing group. CL, clearance; V_d1_, volume of distribution in compartment 1; V_d2_, volume of distribution in compartment 2; AUC, area under the curve; K10, the elimination rate from the central department.

### Oral administration of taurine to male Sprague‐Dawley rats

**Figure 6:**
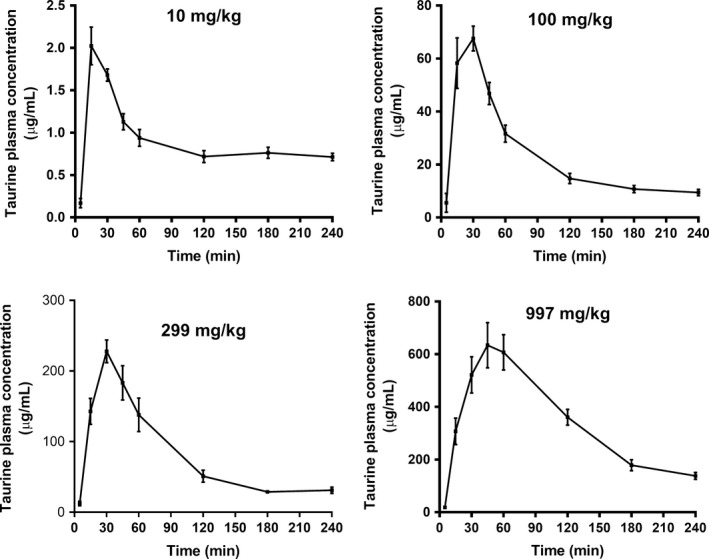
Taurine PK curves after oral administration of taurine to male Sprague‐.Taurine plasma concentration versus time profiles following oral administration of [^3^H]‐taurine (40 *μ*Ci/kg) and, 10 mg/kg, 100 mg/kg, 299 mg/kg or 997 mg/kg taurine. Data is presented as mean ± SEM of measurements from four rats per dosing group.

**Figure 7:**
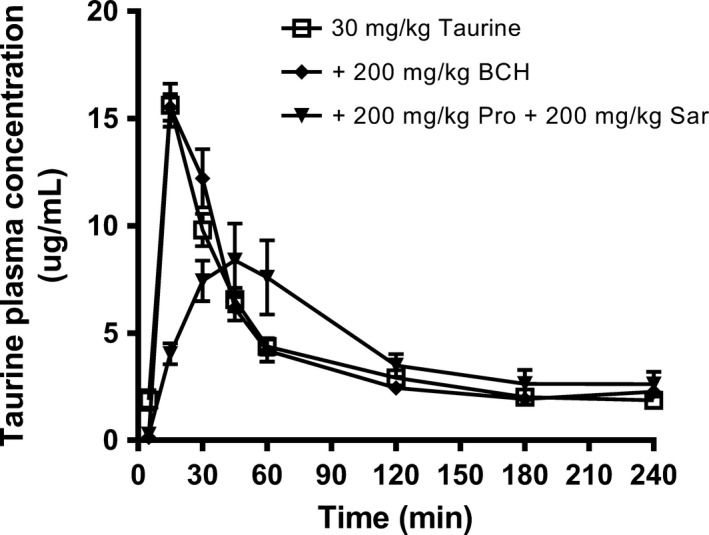
Taurine PK curves after oral administration of 30 mg/kg taurine to male Sprague‐Dawley rats in the presence of amino acids. Taurine plasma concentration versus time profiles after oral co‐administration of [^3^H]‐taurine (40 *μ*Ci/kg) and taurine (30 mg/kg) (control) or with 200 mg/kg BCH or a combination of 200 mg/kg Pro and Sar. Data is presented as mean ± SEM of measurements from 4 to 8 rats per dosing group.

Taurine was administered orally to male Sprague‐Dawley rats in doses from 10 to 997 mg/kg. The plasma concentration versus time profiles are presented in Figure 6 and for 30 mg/kg taurine in Figure 7. The pharmacokinetic parameters (*t*_max_, *C*_max_, AUC_0‐tlast_ and *k*_e_) obtained from the noncompartmental analysis are shown in Table 4. After a single orally administered dose of taurine, the time to reach maximal plasma concentration increased from 15 min at the two lowest doses to 30 min at the intermediate doses (not significantly with the employed test) and finally significantly to 45 min at the highest dose. The maximal plasma taurine concentration was found to be in the range of 0.1–643.4 *μ*g/mL. When the AUC was normalized to the dose, differences were observed in the AUC over dose as shown in Table 4 with the ratio increasing as a function of increased doses.

**Table 4:** Pharmacokinetic parameters of taurine after oral administration to male Sprague‐Dawley rats

Taurine dose (mg/kg)	*t*_max_ (min)	*C*_max_ (*μ*g/mL)	AUC_0‐tlast_ (*μ*g·min/mL)	AUC_0‐∞_/dose (*μ*g·min/mL pr. mg/kg)	*k*_e_ (1/min)
10	15.0 [15.0; 26.25]	2.0 ± 0.2	213 ± 13	21 ± 1.3	0.004 ± 0.001
30	15.0 [15.0; 26.25]	15.7 ± 2.4	1116 ± 134	37 ± 4.5	0.007 ± 0.001
100	30.0 [30.0; 30.0]	70.0 ± 6.5	5396 ± 601	54 ± 6.0	0.010 ± 0.0001
299	30.0 [30.0; 30.0]	227.8 ± 16.0	18400 ± 2001	62 ± 6.7	0.010 ± 0.001
997	45.0 [45.0; 56.25]	643.4 ± 81.0	79118 ± 6692	79 ± 6.7	0.009 ± 0.0009

Rats were orally administered 40 *μ*Ci/kg [^3^H]‐taurine and taurine in various doses. The pharmacokinetic parameters *C*_max_, AUC and *k*_e_ are expressed as mean ± SEM, while *t*_max_ is expressed as the median [Q_1_; Q_3_] (25% and 75% percentile) of measurements from 4 rats per dosing group. *t*_max_, time to reach maximum concentration; *C*_max_, the maximum concentration; *k*_e_, elimination rate constant.

### Oral taurine pharmacokinetics in the presence of BCH, sarcosine, and proline

The pharmacokinetic profile after oral administration of taurine (30 mg/kg) was compared to the oral co‐administration of taurine in the presence of 200 mg/kg BCH or a combination of 200 mg/kg Pro and Sar (Fig. 7 and Table 5). To administer the dose of 30 mg/kg taurine, the animals were given a solution of 30 mg per 10 mL, which corresponded to a taurine concentration of 24 mmol/L. The initial concentration in the gastro‐intestinal system of the rats was thus assumed to be approximately 24 mmol/L. Co‐administration of the PAT1 substrates, Pro and Sar, significantly altered the taurine PK profile by reducing the maximal plasma concentration, *C*_max_, and increasing the time to reach this, *t*_max_, whereas the overall exposure (AUC) remained unchanged. The taurine plasma concentration versus time profiles after co‐administration of the non‐PAT1 ligand BCH was similar to the taurine profile in the absence of co‐administered amino acid.

**Table 5:** Pharmacokinetic parameters of 30 mg/kg taurine after oral co‐administration with BCH or Pro and Sar to male Sprague‐Dawley rats

Taurine (mg/kg)	BCH (mg/kg)	Sar (mg/kg)	Pro (mg/kg)	*t*_max_ (min)	*C*_max_ (*μ*g/mL)	AUC_0‐tlast_ (*μ*g·min/mL)
30	–	–	–	15.0 [15.0; 15.0]	15.6 ± 1.0	1181 ± 62
30	200	–	–	15.0 [15.0; 15.0]	15.5 ± 0.6	1234 ± 91
30	–	200	200	37.5* [30.0; 45.0]	8.5 ± 1.7*	1324 ± 153

Rats were orally administered 30 mg/kg taurine alone or with BCH (200 mg/kg) or a combination of proline and sarcosine (200 mg/kg). The pharmacokinetic parameters *C*_max_ and AUC are expressed as mean ± SEM, while *t*_max_ is expressed as the median [Q_1_; Q_3_] (25% and 75% percentile) of measurements from 4 to 8 rats per dosing group. * significantly different from administration of taurine alone.

## Discussion

In this study, we investigated intestinal taurine absorption and taurine pharmacokinetics at different doses, and investigated taurine disposition after intravenous administration. The mechanism behind intestinal taurine absorption was investigated using an in vivo co‐administration approach and in vitro experiments in Caco‐2 cells. The main findings were that oral taurine absorption was not saturable with increasing taurine doses, and that the time to reach the maximal plasma concentration increased with dose and after administration with PAT1 substrates. Since the absorption could be delayed and reduced with PAT1 substrates this all point toward a role of PAT1 in mediating the intestinal absorption of taurine. The implication is that the intestine is capable of absorbing large amount of taurine after a single dose or intake. It appears, that 30 mg/kg taurine could be a standard substrate dose for pharmacokinetic studies investigating changes in intestinal PAT1 transport activity after physiological or pharmacological regulation or for investigating the possibility of drug/drug or drug/nutrient interactions at the level of intestinal transport.

### Taurine absorption was facilitated by PAT1 in vitro

The intestinal transport mechanism of taurine was investigated in Caco‐2 cells. Firstly, the uptake of taurine into Caco‐2 cells was investigated at neutral extracellular pH (pH 7.4) and slightly acidic pH (pH 6.0). Taurine transport via TauT is sodium‐ and chloride‐dependent and proton‐independent, whereas transport via PAT1 is proton‐dependent and sodium‐ and chloride‐independent (Anderson et al. 2009). We therefore used pH, taurine concentration and PAT1 and TauT inhibitors to distinguish between PAT1 and TauT mediated uptake, while maintaining physiological concentrations of sodium and chloride. Anderson et al. (2009) have earlier investigated taurine uptake in Caco‐2 cells using a different strategy where the PAT1‐mediated taurine uptake was measured at an extracellular pH of 5.5 in the absence of sodium and TauT‐mediated transport was shown by the reduced uptake at pH 7.4 in the absence of sodium or chloride ions. Taurine uptake, in our study, under neutral pH condition showed saturable kinetics with a *K*_m_‐value of 5.6 *μ*mol/L and under slightly acidic (pH 6.0) conditions with a sodium‐containing buffer we found a *K*_m_‐value of 7.1 mmol/L. In the absence of sodium in the uptake buffer and at pH 5.5 Anderson found a *K*_m_‐value of 10.1 mmol/L. Considering the different *K*_m_‐values of taurine and the difference in driving force, we attribute the uptake measured at pH 7.4 to TauT only, and could therefore estimate that at slightly acidic pH of 6.0, PAT1 was the dominating transporter at taurine concentrations above 15 *μ*mol/L. Anderson et al. (2009) have previously shown similar conclusions in Caco‐2 cells estimated as differences in the chloride‐dependent uptake of taurine at an extracellular pH of 6.5.

To further understand the mechanism for intestinal taurine absorption, we measured the uptake of taurine at 52 nmol/L and 100 *μ*mol/L at neutral and slightly acidic pH in the presence of different concentrations of BCH, Sar, Pro as well as IAA. At neutral pH, with 52 nmol/L of taurine, the uptake was via TauT, and was not inhibited by 10 mmol/L of BCH, Pro or Sar, but inhibited by 20 mg/mL BCH and Sar. Since 10 mmol/L BCH and Sar had no effect on taurine uptake, the inhibitory effect at the higher concentration was likely caused by the high osmolarity of the solutions. It is important to note that the uptake studies were conducted on cells cultured on plastic ware and not on filter support. The consequence is that the cells are not exposed to a basolateral medium for equilibration and solute transport. Therefore, the osmotic effect is less on filter‐grown cells and in situations were more solute is transported into the cells, as under slightly acidic conditions were PAT1 transports taurine. A recently identified ligand of TauT, IAA (Valembois et al. 2017), inhibited the taurine uptake at neutral pH and slightly acidic pH at 52 nmol/L taurine, but not at 100 *μ*mol/L, pH 6.0 where PAT1 was dominating. It was an interesting finding that IAA may be a selective TauT inhibitor, since most substrates of TauT such as, for example, taurine, GABA, and *β*‐Ala are also substrates of PAT1 (Frølund et al. 2013). However, this need further clarification using PAT1‐expression systems. The inhibitor studies showed that 20 mg/mL BCH is not likely to inhibit taurine transport via TauT or PAT1, and that 20 mg/mL Sar and Pro will inhibit uptake via PAT1 without any effect on TauT mediated uptake. To confirm that cellular influx via PAT1 resulted in actual transepithelial intestinal transport, the transport of taurine across Caco‐2 cell monolayers was measured. Under conditions designed to evaluate TauT mediated transport (Fig. 7A) of taurine, 20 mg/mL BCH, Sar or Pro did not alter transepithelial taurine transport. Under conditions aimed at evaluating PAT1 mediated transport, BCH did not affect permeability, whereas 20 mg/mL Sar and Pro clearly reduced the transepithelial transport of taurine. Thus, influx of taurine via PAT1 resulted in facilitating transepithelial transport and inhibition of PAT1 reduced this transport.

### Taurine absorption and exposure after oral and intravenous administration

The pharmacokinetic profile after intravenous administration could be described by a two‐compartment model with first‐order rate constants and elimination from the central compartment. The different doses did not result in significant differences in taurine clearance or in dose‐normalized exposure. The clearance in this study was approximately 9–18 mL/min/kg (0.56–1.1 L/h/kg), whereas the clearance in Sprague‐Dawley rats dosed with 20 mg/kg was reported to be 0.13–0.15 L/h/kg (Tang et al. 2014), thus lower than in the present study. In beagle dogs a clearance of 0.20 L/h/kg have been reported (Yu et al. 2013), which was also lower than the values obtained here. In the present study, a compartmental analysis was used for the evaluation of the plasma data, whereas the two mentioned studies used noncompartmental analysis.

Taurine was furthermore dosed orally in increasing doses ranging from 10 to 997 mg/kg. As dose increased, AUC_0‐tlast_ increased as well, but a linear correlation was not observed. In fact, the AUC_0‐tlast_ increased more with increasing dose. If intestinal absorption is saturated a decrease in dose normalized AUC is expected, as clearly shown with the drug substance gabapentin, which is a substrate for an intestinal carrier with a low transport capacity (Larsen et al. 2015). For gabapentin, a clear dose‐dependent absorption in Sprague‐Dawley rats was observed with a decreasing AUC over dose with increasing doses due to saturation of the absorption step (Larsen et al. 2015). IV administration of three different doses of taurine did not reveal any changes in clearance as discussed above, hence a possible explanation for the lack of dose linearity could be saturation of the accumulation of taurine in tissues such as kidney, brain and eye, which would result in an increased taurine plasma concentration. Another explanation could be a limitation of elimination, however, when examining the elimination rate constants, estimated via a noncompartmental analysis, an increase was not observed as the dose increased. Thus, a limitation in elimination most likely would not be the cause of the lack of dose proportionality. Sved et al. (2007) have previously presented pharmacokinetic profiles, where 30 mg/kg and 300 mg/kg taurine were administered orally to male Sprague‐Dawley rats. The AUC values obtained were 236 *μ*g·h/mL and 1760 *μ*g·h/mL, respectively. Compared to the AUC_0‐tlast_ values obtained in this study of 1116 ± 134 *μ*g·min/mL (30 mg/kg) and 18400 ± 2001 *μ*g·min/mL (299 mg/kg), the values presented by Sved et al. (2007) were larger. An explanation could be in the pharmacokinetic analysis, where Sved et al. have extrapolated AUC to infinity due to a longer sampling period. The pharmacokinetic profile of taurine has also been examined after oral administration in humans (Ghandforoush‐Sattari et al. 2010). Taurine was administered in a dose of 4 g, correlating to 50 mg/kg with a median volunteer weight of 79.5 kg. The elimination rate constant was comparable to the value presented in this study, but *C*_max_, *t*_max_, and AUC_0‐tlast_ estimated in humans were all higher, when compared to doses of 30 and 100 mg/kg in rats. Collectively, the presented study has demonstrated that the disposition of taurine was not saturable at IV doses and plasma concentrations shown, and that oral taurine absorption was not saturable with increasing dosing, yet had increased time to reach maximal plasma concentration with increasing dose. The latter is an indication of carrier‐mediated absorption via a carrier with a high transport capacity and an expression along the length of the intestine.

Taurine was administered to male Sprague‐Dawley rats at a dose of 30 mg/kg, corresponding to an initial intestinal concentration of 24 mmol/L, in combination with 20 mg/mL BCH, Pro and Sar to male Sprague‐Dawley rats. The dose chosen was comparable to the concentration found in common “High Energy” drinks, and clearly much higher than the concentration required to saturate TauT mediated transport. The presence of BCH, which is not a substrate of TauT or PAT1 in vitro and does not affect PAT1 function in vivo in rats (Nohr et al. 2014c), did not have any significant effect on any of the pharmacokinetic parameters AUC_0‐tlast_, *t*_max_, and *C*_max_. However, when taurine was co‐administrated with proline and sarcosine, both PAT1 substrates, a significant increase in *t*_max_ and a decrease in *C*_max_ was observed, without an effect on AUC_0‐tlast_. We have previously shown a similar pattern for gaboxadol absorption in rats and dogs, and for vigabatrin in rats (Larsen et al. 2009; Broberg et al. 2012; Nohr et al. 2014c), and attributed this to carrier‐mediated intestinal absorption via PAT1. It is known, that several factors have an impact on the gastric emptying rate. Particularly the intake of high‐calorie meals decreases the gastric emptying rate with a near‐to linear correlation (Calbet and MacLean 1997). The effect observed on *C*_max_ and *t*_max_, after co‐administration of taurine with proline and sarcosine, could therefore also reflect the influence on the gastric emptying rate and not be related to amino acid mediated inhibition of absorption via PAT1. However, BCH did not alter the PK profile of taurine, and we have previous shown that that 200 mg/kg sarcosine do not alter the gastric emptying rate in rats (Nohr et al. 2014c). High doses of amino acids were used since the intestinal transport capacity of PAT1 is difficult to saturate due to the expression along the small intestine and the high transport capacity of PAT1 (Broberg et al. 2012; Nohr et al. 2015) that due to gastrointestinal transit enables absorption of high doses.

In conclusion, the presented study has shown that taurine disposition in rats was unaltered at the doses investigated. After oral taurine administration, it was not possible to saturate taurine absorption, suggesting a large intestinal absorptive capacity. In vitro and in vivo data in this study supported that the high taurine clearance from the intestine may be explained by the intestinal transport of taurine via the low‐affinity high‐capacity amino acid transporter, PAT1.

## Compliance with ethical standards

All studies conducted here comply with ethical standards.

## Ethical approval

All procedures performed in studies involving animals were in accordance with the ethical standards of the institutional animal ethics committee in accordance with Danish law regulating animal experiments and in compliance with EC Directive 2010/63/EU and the NIH guidelines on animal welfare.

## Conflict of Interest

The authors declare that they have no conflict of interest.
